# Enhanced Anti-Tumor Response Elicited by a Novel Oncolytic Pseudorabies Virus Engineered with a PD-L1 Inhibitor

**DOI:** 10.3390/v16081228

**Published:** 2024-07-31

**Authors:** Guangtao Xiang, Mengdong Wang, Pu Wang, Rifei Li, Chao Gao, Yue Li, Xinxin Liang, Yun Liu, Aotian Xu, Jun Tang

**Affiliations:** 1 College of Veterinary Medicine, China Agricultural University, Beijing 100193, China; 2 Cytovaxis Biotechnologies Inc., Guangzhou 510760, China

**Keywords:** oncolytic virus, PRV, mouse melanoma, iPD-L1

## Abstract

Oncolytic viruses combined with immunotherapy offer significant potential in tumor therapy. In this study, we engineered a further attenuated pseudorabies virus (PRV) vaccine strain that incorporates a PD-L1 inhibitor and demonstrated its promise as an oncolytic virus in tumor therapy. We first showed that the naturally attenuated PRV vaccine strain Bartha can efficiently infect tumor cells from multiple species, including humans, mice, and dogs in vitro. We then evaluated the safety and anti-tumor efficacy of this vaccine strain and its different single-gene deletion mutants using the B16-F10 melanoma mouse model. The *TK* deletion strain emerged as the optimal vector, and we inserted a PD-L1 inhibitor (iPD-L1) into it using CRISPR/Cas9 technology. Compared with the control, the recombinant PRV (rPRV-iPD-L1) exhibited more dramatic anti-tumor effects in the B16-F10 melanoma mouse model. Our study suggests that PRV can be developed not only as an oncolytic virus but also a powerful vector for expressing foreign genes to modulate the tumor microenvironment.

## 1. Introduction

Immunotherapy is a type of cancer treatment that enhances the immune system’s ability to fight against cancer, which has revolutionized the field of cancer treatment and rejuvenated tumor immunology [1]. Several types of immunotherapies have been developed, including immune checkpoint inhibitors (ICIs), T-cell transfer therapy, treatment vaccines, and immune system modulators [2,3,4]. ICIs work by blocking suppressive receptors on T cells, allowing them to kill cancer cells more efficiently. Well-studied checkpoint proteins include programmed cell death 1 (PD-1), programmed cell death 1 ligand 1 (PD-L1), and cytotoxic T lymphocyte-associated antigen 4 (CTLA-4) [5]. So far, the most widely used immunotherapeutic agents are blocking antibodies, such as Ipilimumab, Nivolumab, and Atezolizumab, which target these immune inhibitory receptors [6]. Despite the success of ICIs, only about 10–40% of patients with certain tumors benefit from checkpoint blockade therapy [7], due to limited immune cell infiltration and the immunosuppressive tumor microenvironment [8]. To overcome this challenge, various combination strategies have been developed [9,10,11]. Oncolytic viruses are a promising approach, exhibiting distinctly different anti-tumor activity compared to other anticancer therapies [12].

Oncolytic viruses represent a new class of therapeutic agents that infect tumor cells, leading to the destruction of infected cells and induction of systematic anti-tumor immunity [13,14]. Initially, the development of oncolytic viruses primarily focused on their ability to directly infect and lyse tumor cells. The general downregulation of type I interferon (IFN) and other antiviral signaling pathways in cancer cells make them more vulnerable for oncolytic virus infection and replication. With advances in genetic engineering, oncolytic viruses are often manipulated through gene deletions or insertions to improve their target specificity, selective replication and oncolytic ability, as well as their safety. Up to now, the critical importance of immunoreactive response during tumor lysis following oncolytic virus infection has been recognized [15]. During oncolytic virus infection, viral elements such as viral capsids, DNAs, RNAs, and proteins act as pathogen-associated molecular patterns (PAMPs) and are recognized by the host immune system. Moreover, oncolytic viruses can also induce various forms of immunogenic cell death (ICD), including pyroptosis, necroptosis and immunogenic apoptosis, by inducing endoplasmic reticulum (ER) stress [16]. The dying host cells release hallmark immunostimulatory damage-associated molecular patterns (DAMPs). These PAMPs and DAMPs are sensed by pattern recognition receptors (PRRs), such as the cyclic GMP-AMP synthase (cGAS) and Toll-like receptors (TLRs), establishing a proinflammatory microenvironment [17]. This environment stimulates the production of proinflammatory cytokines (such as type I IFNs, Tumor Necrosis Factor Alpha (TNF-α), granulocyte macrophage colony-stimulating factor (GM-CSF), interleukin (IL)-1β, IL-6), and chemokines (such as C-C motif chemokine ligand 2 (CCL2), CCL3, C-X-C motif chemokine ligand 9 (CXCL9), and CXCL10), turning the “cold” tumor into an inflamed, immunologically “hot” tumor [18].

Various virus species are under development as oncolytic agents, including the adenovirus, herpesvirus, vaccinia virus, Newcastle disease virus (NDV), and measles virus, among others [15]. In 2015, Talimogene laherparepvec (T-VEC) developed from type 1 herpes simplex virus (HSV-1) was approved by United States Food and Drug Administration as an oncolytic virus to treat patients with metastatic melanoma, marking a milestone in the field of oncolytic virotherapy. In 2021, G47Δ was approved by Japan as the world’s first OV treatment for malignant glioma. G47Δ is based on HSV-1 with the deletion of the *ICP34.5* and *ICP47* genes and inactivation of the *ICP6* gene to further enhance tumor specificity. However, there are challenges associated with oncolytic virus therapy, such as the acidic conditions and hypoxic tumor microenvironments, a dense extracellular matrix (ECM), and recognition and elimination by the immune system. Additionally, due to the high heterogeneity and complexity of tumors and the different tropism of each oncolytic virus, it is necessary to develop more oncolytic virus therapies. Herpesviruses possess several advantageous qualities for serving as oncolytic agents, including their large coding capacity, high viral titers, extensive molecular virology data, and the ability to reinfect hosts multiple times [19].

The pseudorabies virus (PRV) is a swine pathogen classified within the alpha-herpesvirinae subfamily and the Varicellovirus genus [20]. While swine serve as the natural host and reservoir of PRV, the virus can also infect numerous other mammals, including carnivores, rodents, and ruminants [21,22]. The PRV possesses a double-stranded genomic DNA of approximately 143 kb containing at least 72 genes [23], including virulence-related genes and many non-essential genes. Deletion of virulence genes significantly attenuates PRV, making it suitable as a vaccine strain. Bartha is one of the most widely used vaccine strains. It is a naturally attenuated PRV, in which a segment of DNA encompassing the virulent genes *gE*/*gI* is deleted through a series of passages of a field strain of PRV. The Bartha strain has also been utilized as a vaccine vector due to its large PRV genome, which allows the insertion of exogenous genes, making PRV a valuable vector for expressing major antigens from different swine pathogens [24]. The viral thymidine kinase (*TK*) gene is another important virulence gene often deleted to attenuate viruses. Attenuated PRV is safe for humans but can efficiently infect human cancer cells. Recently, Wang et al. tested the tumor tropism of a live-attenuated swine pseudorabies virus (PRV-LAV), derived from the PRV vaccine strain HB2000 (with deletion of *gG*, *gE*, and *TK*), on the viability of normal human cells and 38 representative cancer cell lines. The results showed that PRV-LAV is sufficiently safe and exhibits cancer-selective cytotoxic activity [25].

In this study, we evaluated the anti-tumor activities of attenuated PRV both in vitro and in vivo. We demonstrated that the Bartha strain can efficiently infect and lyse multiple tumor cell lines from different species. Further attenuated Bartha strains, including those with individual deletions of *TK*, *US3*, or *UL50*, showed improved safety and significant anti-tumor activity in vivo, with the *TK* deletion strain (rPRV-ΔTK) exhibiting the best results. Insertion of a PD-L1 inhibitor into rPRV-ΔTK further enhanced the anti-tumor activity of the virus in a mouse model. Our results suggest that attenuated PRV has the potential to be developed into a potent oncolytic virus capable of expressing immune regulatory genes to further enhance anti-tumor activities.

## 2. Materials and Methods

### 2.1. Viruses and Cells

In the PRV Bartha strain, three single-gene deletion mutants based on Bartha strain rPRV-ΔTK, rPRV-ΔUS3, and rPRV-ΔUL50 have been described previously [26,27]. The recombinant virus with inactivation of the genes *TK* and *EP0*, and red fluorescent protein (RFP) insertion at *EP0* site, rPRV-ΔTK/EP0-RFP (rPRV-RFP) from the laboratory collection. The mouse pancreatic cancer cells Panc02, and mouse melanoma cells B16-F10, were grown in Roswell Park Memorial Institute (RPMI) 1640 medium (M&C gene Technology, Beijing, China) supplemented with 10% filtered, heat-inactivated fetal bovine serum (FBS) (TransGen Biotech, Beijing, China) and 100 µg/mL penicillin (M&C gene Technology, Beijing, China). PK15 (porcine kidney 15), B-CMT (a canine mammary tumor cell line established and stored by our lab [28]), human normal cell IMR-90, human tumor cell HeLa, Hep2, HepG2, A549, U-2OS, HCT116 cells were cultured in Dulbecco’s Modification of Eagle’s Medium (DMEM) (M&C gene Technology, Beijing, China) supplemented with 10% FBS and 100 µg/mL penicillin. All those cells were cultured in a constant temperature incubator at 37 °C with 5% CO_2_ for passaging.

### 2.2. Virus Titration

PK15, Panc02, and B-CMT cells were infected with the indicated viruses at a MOI of 1 and then incubated at 37 °C for 2 h in an incubator containing 5% CO_2_. The supernatant was discarded, and a fresh DMEM medium was added. Then, we collected the supernatants at the indicated time points for virus titer determination. Then, 50% tissue culture infective doses (TCID_50_) were determined by 50% endpoint dilution determination using the Reed and Muench method to calculate the virus titer in PK15 cells.

### 2.3. Cell Viability Assay

In total, 2 × 10^5^ of human cancer cells and normal cell IMR-90 were seeded onto 96-well plates and cultured for 24 h. When cells reached 80% confluence, they were infected with Bartha in DMEM medium at a specific multiplicity of infection (MOI) and incubated at 37 °C. At 72 h and 96 h, 10 µL of Cell Counting Kit-8 (CCK-8) solution (Solarbio, CA1210, Beijing, China) was added to each well, and the culture plates were incubated for 1–4 h. The absorbance at 450 nm was measured using a microplate reader (Thermo Fisher Scientific, Waltham, MA, USA). The percentage of cell viability was calculated as follows: (OD value of infected cells − OD value of blank)/(OD value of uninfected cells − OD value of blank) × 100%.

B-CMT and Panc02 cells cell viability assays were conducted as previously described. The cells were infected with Bartha at a specific MOI, and cell viability was assessed 24 h post infection.

### 2.4. Western Blotting

To examine the expressions of viral proteins in Panc02 and B-CMT cells infected with Bartha, whole-cell extracts were collected and lysed in a lysis buffer (50 mM Tris-Cl [pH = 8.0], 10 mM dithiothreitol [DTT], 150 mM NaCl, 1% Triton X-100, 1 × complete protease inhibitor cocktail tablet, and 10% glycerol). The lysates were subjected to SDS-PAGE and transferred onto polyvinylidene difluoride (PVDF) membranes; the membranes were subsequently blocked by 5% fat-free milk PBST (phosphate-buffered saline [PBS] with 0.1% Tween 20) and then blotted with the indicated antibodies. The antibodies against PRV US3 and VP5 were described previously [26,27].

### 2.5. Animal Experiment

Female C57BL/6J mice aged 4 to 5 weeks were purchased from the Beijing Vital River Laboratory Animal Technology. A total of 5 × 10^5^ B16-F10 cells in 100 μL PBS were injected subcutaneously into the right groin of the C57BL/6J mice. When tumors reached a size of 50 mm^3^ to 100 mm^3^, all mice were randomly divided into five or three experimental groups of three mice each. The mice were then intratumorally injected with either DMEM or 1 × 10^7^ TCID_50_ (1 × 10^6.5^ TCID_50_ for evaluation of anti-tumor activity of the recombinant strains) of the indicated PRV strains in volumes of 100 μL every other day for a total of three times, unless otherwise indicated. The body weight of each mouse was recorded every 2 to 3 days. Tumors were measured approximately every 2 to 3 days using a digital caliper, and the tumor volumes were calculated using the formula 1/2 × (Length × Width^2^). Mice were euthanized when the tumor diameter exceeded 2 cm or when mice were excessively moribund.

The workflow was the same as previously described. On the 16th day of the experiment, the mice were euthanized, and tumors, brain, lung, and kidney were collected for histological examination or immunofluorescence staining.

### 2.6. Ethics Statement

All animal experiments were performed in strict accordance with the recommendations in the “Guidelines for the Ethics and Use of Laboratory Animals” established by the Ministry of Science and Technology of the People’s Republic of China. The protocol used in this study was approved by the Institutional Animal Ethics and Use Committee of China Agricultural University (Beijing, China; CAU NO.AW31213202-2-1).

### 2.7. Construct the Recombinant Viruses rPRV-iPD-L1

The gene for mouse iPD-L1 (a murine soluble PD-1 extracellular domain fused with an IgG Fc fragment as a PD-L1 inhibitor) was synthesized by Sangon Biotech (Shanghai, China). The donor plasmid [26] was used as vector to construct the new donor plasmid expressing iPD-L1. The primers used were as follows: for the amplification of the vector, forward, 5-TCTGTTAAAGCAA-GCAGGAGACGTGGAAGAAAACCCCGGTCCTATGGCCTCCTCCGAGGAC-3 and reverse, 5-GGTGGCACCGGTAGCGCTAG-3. For the amplification of iPD-L1, forward, 5-AGCGCTACCGGTGCCACCATGTGGGTCCGGCAGGTA-3 and reverse, 5-CTCCTGCTTGCTTTAACAGAGAGAAGTTCGTGGCTCCGGATCCTTTACCCG-GAGTCCGGGA-3 were used. All constructs were verified by sequencing. *EP0* sgRNA1 and sgRNA2 target the amino-terminal region of *EP0*. PRV genomic DNA was extracted and purified from infected cells using SDS-proteinase K extraction as described previously [26,29]. For generation of recombinant viruses, pCDNA3.1 cas9 (1 µg) expressing Cas9, *EP0* sgRNA1 (0.5 µg), *EP0* sgRNA2 (0.5 µg), donor plasmid (0.5 µg), and viral DNA genome (2.5 µg) were co-transfected into PK15 cells using Lipo 2000 (Mei5 Biotechnology, MF135, Beijing, China) according to the manufacturer’s instructions. Two to four days after transfection, when the cytopathic effect (CPE) was observed, the supernatants were collected and then serially diluted by 10^−1^–10^−8^ fold for subcloning the viruses. Ten rounds of purification were performed for single colonies. Rabbit anti-mouse IgG secondary antibody was used to detect iPD-L1, which contains a mouse IgG Fc fragment. The recombinant viruses were confirmed by Western blotting and polymerase chain reaction (PCR) with primers forward, 5-GGAGCATGGCCTCGGTCAC-3 and reverse, 5-GGTGGCACCGGTAGCGCTAG-3. After the purification of amplified DNA, the fragment was sequenced.

### 2.8. Histological Examination

To examine the tumor tissue or mouse organs under a light microscope, we first preserved the samples in 10% formalin at 4 °C for three days. Next, we dehydrated the tissue using alcohol, butanol, and xylene, and embedded it in paraffin. Slices about 4 µm thick were then cut from the paraffin blocks. These sections were deparaffinized, stained with hematoxylin–eosin, and mounted in Vitrogel before observation. The examination was conducted using an upright optical microscope (Nikon, Tokyo, Japan).

### 2.9. Immunofluorescence (IF) Staining

The 4 μm paraffin-embedded mouse melanoma tissue sections were blocked with 3% H_2_O_2_ and 3% BSA after undergoing dewaxing, hydration and antigen retrieval. Multiplexed immunofluorescence staining of CD4 (Rabbit, 1:2000, abcam, Cambridge, UK), CD8 (Rabbit, 1:1000, abcam, UK), and PD-L1 (Rabbit, 1:1000, CST, Boston, MA, USA) was performed according to the manufacturer’s instructions (AiFang biological, 4-Color Multiple fluorescence Kit, Changsha, China). The images were captured using an AKOYA multispectral microscope. 

### 2.10. Statistical Analysis

Data were analyzed using GraphPad Prism 8.0 Software Statistical and analyses were performed by using Student’s *t*-test, one-way analysis of variance (ANOVA), and two-way analysis of variance (ANOVA). Data are expressed as means ± standard error. *p* values ≤ 0.05 were considered statistically significant. *p*-values: (*) < 0.05; (**) < 0.01; (***) < 0.001; (****) < 0.0001.

## 3. Results

### 3.1. PRV Can Infect and Kill Tumor Cells In Vitro

**Figure 1:**
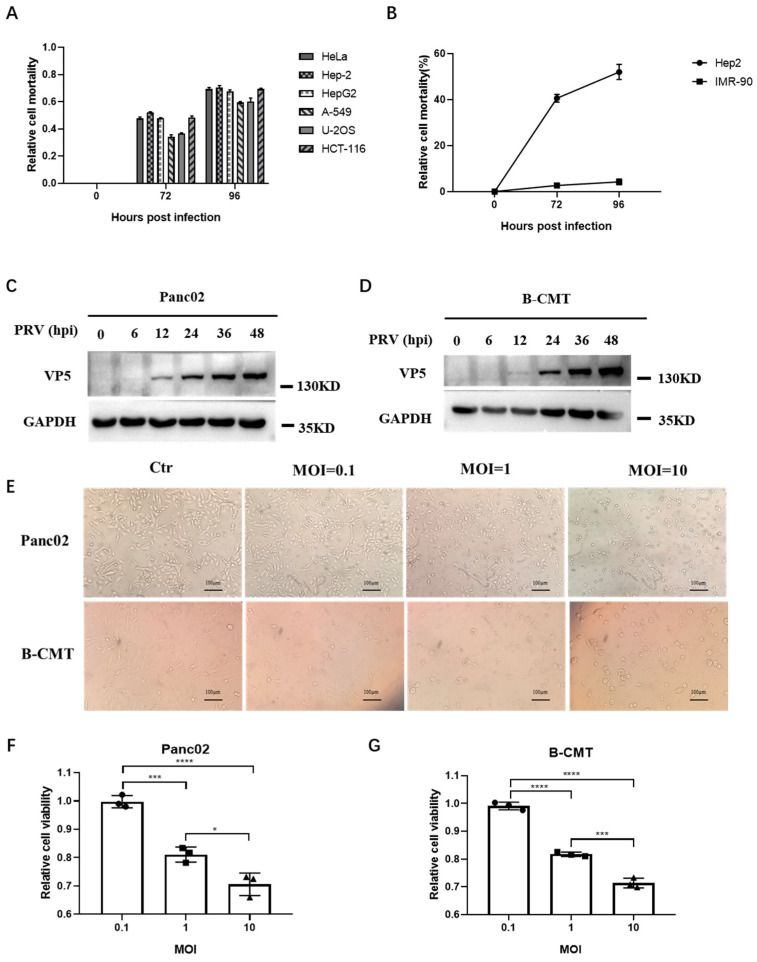
PRV Bartha infects and kills human, mouse, and dog tumor cells. (**A**) Cell viability assays using CCK-8 kit were performed on six representative human tumor cell lines at 72 h and 96 h after PRV Bartha infection (MOI = 1). (**B**) Cell viability assays were performed on the human normal cell line IMR-90 and the human cancer cell Hep2 at 72 and 96 h after exposure to PRV Bartha (MOI = 1). (**C**,**D**) The expression of PRV VP5 was analyzed by Western blot after PRV Bartha infected the mouse tumor cell line Panc02 and the dog tumor cell line B-CMT (MOI = 1). The cell samples were collected as the indicated time points. GAPDH was used as a loading control. (**E**) Cell morphology of Panc02 and B-CMT cells 24 h post infection with PRV Bartha at 0.1, 1, or 10 MOI. (**F**,**G**) PRV Bartha was used to infect Panc02 and B-CMT. Cell viability was assessed using the CCK-8 kit. Data are presented as the mean ± s.d. values (*n* = 3). A one-way analysis of variance (ANOVA) was used to determine the significance of differences between the infection groups. * *p* < 0.05, *** *p* < 0.001, **** *p* < 0.0001. hpi: hours post infection.

To assess PRV’s ability to infect and kill tumor cells across different species, we first infected human cancer cells with the PRV Bartha strain. PRV was shown to kill many types of human tumor cells (Figure 1A). We further compared PRV-induced killing between the human cancer cell line Hep2 and the normal cell line IMR-90, demonstrating that PRV efficiently killed Hep2 while leaving IMR-90 alive (Figure 1B). This suggests that PRV can specifically target and kill human tumor cells. We then infected mouse and dog tumor cells (Panc02 and B-CMT, respectively) with PRV Bartha. Western blotting analysis indicated that PRV successfully infected both cell types (Figure 1C,D). Cell images and viability assays showed that PRV effectively killed both mouse tumor cells (Panc02) and dog tumor cells (B-CMT) in a dose-dependent manner (Figure 1E–G). Collectively, these results suggest that PRV can infect and kill tumor cells across species.

### 3.2. PRV Reduces Tumor Growth Rates and Enhances Survival in an Immunocompetent Model

**Figure 2:**
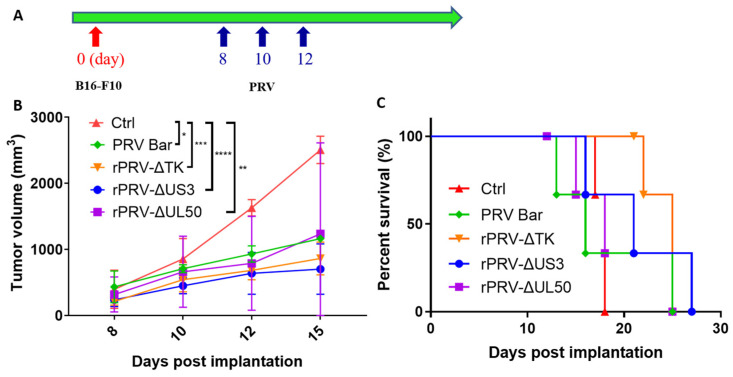
PRV reduces tumor growth rates and enhances survival in an immunocompetent model. (**A**) Workflow for the B16-F10 mouse melanoma tumor model and oncolytic virus treatment. When tumors reached 50 mm^3^ to 100 mm^3^, all mice were randomly divided into five experimental groups of three mice each. The mice were then intratumorally injected with either DMEM, 1 × 10^7^ TCID_50_ of PRV Bartha, or single-gene knockout PRV Bartha strains (rPRV-ΔTK, rPRV-ΔUS3 and rPRV-ΔUL50), in volumes of 100 μL every other day for a total of three times. (**B**) Tumor volume was quantified at the indicated time points (*n* = 3). (**C**) The overall survival of tumor-bearing mice (*n* = 3). PRV Bar: PRV Bartha; rPRV-ΔTK, rPRV-ΔUS3 and rPRV-ΔUL50: deletion mutants of on *TK*, *US3* and *UL50* respectively based on the Bartha strain. *p < 0.05; ** *p* < 0.01; *** *p* < 0.001; **** *p* < 0.0001.

The *TK*, *UL50*, and *US3* genes of PRV are associated with pathogenicity of PRV. *TK* and *UL50* are involved in viral nucleic acid metabolism, while *US3* regulates apoptosis. *US3* and *UL50* also play a role in viral immune evasion. Importantly, single deletions of these genes from Bartha further improve the safety [30,31,32]. To assess the anti-tumoral efficacy of these different single-gene deletion PRV strains in vivo, we monitored tumor growth and survival rates in the C57/6J B16-F10 mouse melanoma model. The mice were randomly divided into five groups (*n* = 3), the tumor-bearing mice were treated with DMEM, PRV Bartha, or single-gene knockout PRV Bartha strains (rPRV-ΔTK, rPRV-ΔUS3 or rPRV-ΔUL50), every other day via intratumoral injection for a total of three times when the average tumor volume reached 50–100 mm^3^ (Figure 2A). As expected, all PRV strains inhibited tumor growth, with rPRV-ΔTK and rPRV-ΔUS3 strains exhibiting better effects (Figure 2B). Considering the overall survival data, rPRV-ΔTK was even better (Figure 2C). These data demonstrate that all PRV strains examined exhibit anti-tumoral efficacy in vivo, with rPRV-ΔTK emerging as the most promising oncolytic virus candidate.

### 3.3. Construction of the Recombinant PRV Expressing iPD-L1 by CRISPR/Cas9

**Figure 3:**
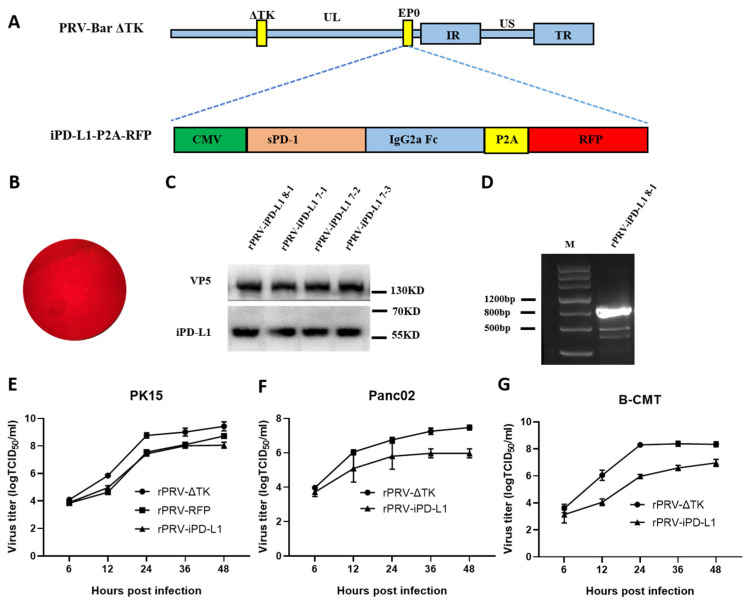
Construction of the recombinant PRV strain expressing the PD-L1 inhibitor (rPRV-iPD-L1) with CRISPR/Cas9. (**A**) The scheme of rPRV-iPD-L1 construction. (**B**) Microphotographs of plaques exhibiting red fluorescence after PK15 cells were infected with the rPRV-iPD-L1 recombinant strain. (**C**) Western blotting analysis of cell lysates from PK15 cells infected with rPRV-iPD-L1. (**D**) Confirmation of iPD-L1 insertion by PCR. (**E**–**G**) PK15, Panc02, and B-CMT were infected with rPRV-iPD-L1 at an MOI of 1, and supernatants were collected at different time points for virus titration. sPD-1: a murine soluble PD-1 extracellular domain.

To enhance anti-tumor activity, we inserted a PD-L1 inhibitor (iPD-L1) driven by the human cytomegalovirus (hCMV) promoter into the *EP0* locus of PRV-ΔTK (Figure 3A). We named the new recombinant virus rPRV-iPD-L1. iPD-L1 expresses a fusion consisting of a murine soluble PD-1 extracellular domain and an IgG Fc fragment, which has been shown to strongly inhibit the binding between PD-1 and PD-L1 [33]. The donor plasmid expresses iPD-L1 linked to RFP via a P2A (porcine teschovirus-1 2A) self-cleaving peptide. The plasmids of the CRISPR/Cas9 system, the donor plasmid expressing iPD-L1 and the genomic DNA of PRV-ΔTK, were co-transfected into PK15 cells. Plaques exhibiting red fluorescence (Figure 3B) were then screened and purified over ten rounds. Western blotting analysis confirmed the expression of iPD-L1 in infected cells (Figure 3C). PCR showed that the iPD-L1 DNA was inserted into the genome of PRV (Figure 3D). Agarose gel electrophoresis of the PCR product exhibited a band size consistent with the expected size, indicating successful amplification. The band was then sent for sequencing, confirming the successful construction of rPRV-iPD-L1. rPRV-iPD-L1 showed a slower replication rate than rPRV-ΔTK in PK15 cells (Figure 3E). Similar results were observed in mouse and dog tumor cells Panc02 and B-CMT (Figure 3F,G).

### 3.4. Anti-tumor Activity of the Recombinant Strain rPRV-iPD-L1 In Vivo

**Figure 4:**
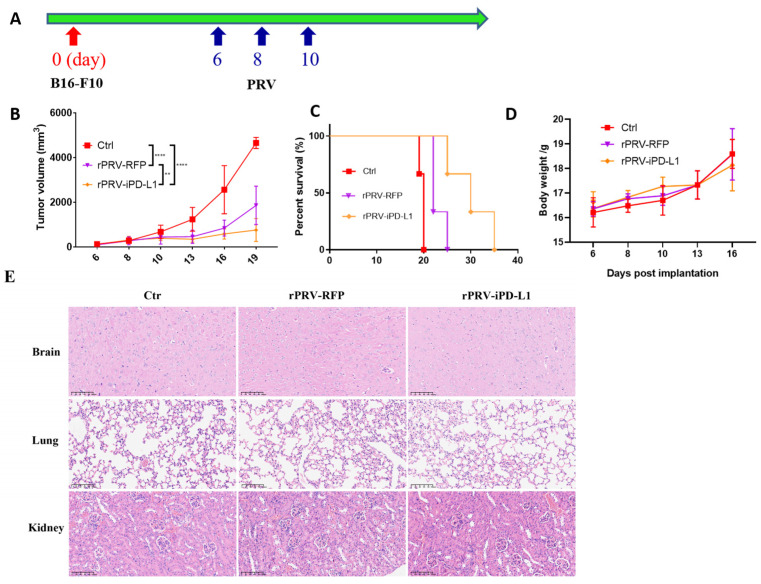
Anti-tumor activity of the recombinant strain rPRV-iPD-L1 in vivo. (**A**) Workflow for the B16-F10 melanoma tumor model and oncolytic virus treatment. (**B**) After tumors reached 50 mm^3^ to 100 mm^3^, all mice were randomly divided into three groups of three mice per group. Each mouse was intratumorally injected with either DMEM, 1 × 10^6.5^ TCID_50_ of rPRV-iPD-L1, or rPRV-RFP in volumes of 100 μL every other day for a total of three times. Tumor volumes were measured approximately every 2 to 3 days and are presented as *n* = 3. (**C**) The overall survival rate of C57/6J mice bearing B16-F10 melanoma treated with rPRV-iPD-L1, rPRV-RFP, or the control group. (**D**) The body weight of each group after treatment. (**E**) On the 16th day, brain, lung, and kidney samples were taken for HE staining in each group. The image displayed represents one mouse from each group, with 20× magnification (Scare bar = 100 μm). ** *p* < 0.01; **** *p* < 0.0001.

We then evaluated the anti-tumor activity of rPRV-iPD-L1 using a B16-F10 melanoma syngeneic transplant mouse model, in comparison with iPD-L1 un-expressing control virus. When tumors reached 50 mm^3^ to 100 mm^3^, the tumor-bearing mice were divided randomly into three groups (*n* = 3), then they received intratumoral injections of rPRV-iPD-L1, rPRV-RFP, or DMEM according to the schedule (Figure 4A). The tumor volumes were measured approximately every 2 to 3 days. The results showed that rPRV-iPD-L1 was more potent in inhibiting B16-F10 tumor growth (Figure 4B). The survival curve (Figure 4C) also supported the observation. rPRV-iPD-L1 appeared to be safe to use as no difference in body weight between the different groups was observed (Figure 4D) and pathological analysis also did not reveal any abnormalities in the brains, lungs, or kidneys in rPRV-iPD-L1- and rPRV-RFP-treated mice (Figure 4E). In addition, no evidence of viremia was detected in rPRV-iPD-L1- and rPRV-RFP- treated mice. These results indicate that both rPRV-RFP and rPRV-iPD-L1 are safe as oncolytic viruses, with rPRV-iPD-L1 exhibiting enhanced anti-tumor activity.

### 3.5. PRV Treatment Increases the Infiltration of Lymphocytes within the Tumor Microenvironment

**Figure 5:**
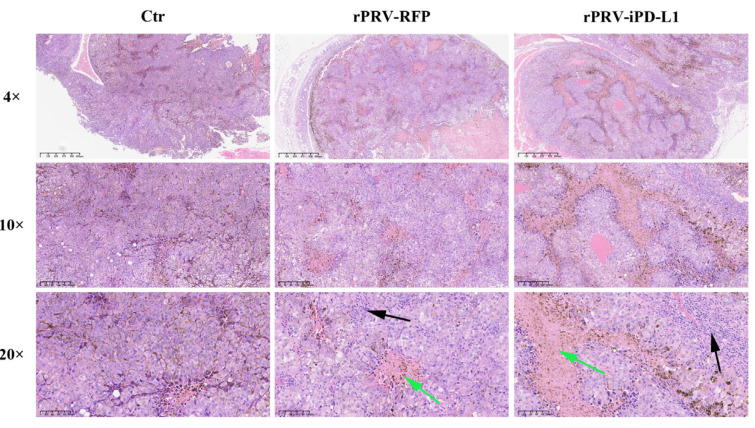
Histopathological examination of tumor tissues from mice treated with PRV. The image displayed represents one mouse from each group and is shown at 4×, 10×, and 20× magnification (Scale bar = 100 μm). The green arrows indicate focal necrosis of tumor cells, a result of treatment with PRV. The black arrows indicate infiltration of tumor tissue by inflammatory cells, a common response to infection or injury.

On the 16th day of the experiment (following the workflow as in Figure 4A), we evaluated the pathological differences in tumor areas in vivo between the control and the rPRV-iPD-L1- or rPRV-RFP- treated mice. The results showed that the tumor areas in rPRV-RFP- treated mice displayed evident focal necrosis, which was exacerbated in the rPRV-iPD-L1- treated group. The areas of focal necrosis were increased, and the overall structure of the tumor tissue appeared unconsolidated. In contrast, the control group treated with DMEM showed no significant lymphocyte infiltration or cell necrosis in the tissue (Figure 5). These findings suggest that PRV treatment enhances focal necrosis and infiltration of lymphocytes within the tumor microenvironment.

**Figure 6:**
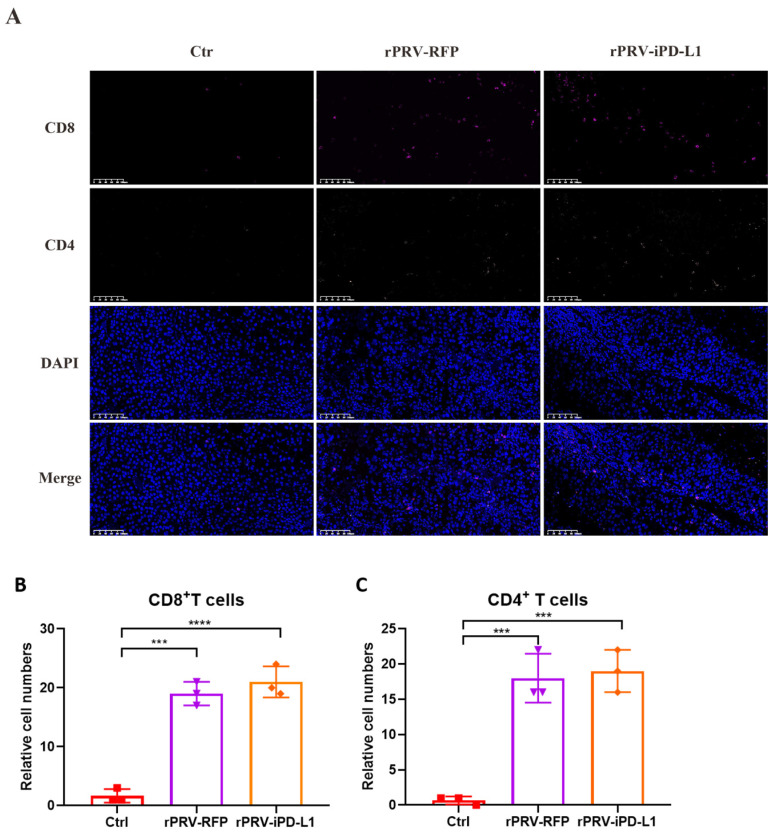
Immunofluorescence of CD4^+^ and CD8^+^ T cells within the tumor microenvironment. (**A**) Immunofluorescence staining of CD4^+^ and CD8^+^ T cells was performed in each group. The image displayed represents one mouse from each group and is shown at 20× magnification (Scale bar = 100 μm). (**B**, **C**). Relative cell numbers of CD4^+^ and CD8^+^ T cells were counted. Data are presented as the mean ± s.d. values (*n* = 3). Statistical analysis was performed using one-way analysis of variance (ANOVA). *** *p* < 0.001; **** *p* < 0.0001.

We further stained CD4^+^ and CD8^+^ T cells in the tumor microenvironment of different groups of mice. Immunofluorescence analysis demonstrated a significantly elevated presence of CD4^+^ and CD8^+^ T cells in the tumor tissues of mice treated with rPRV-iPD-L1 and rPRV-RFP (Figure 6A–C). These results suggest that PRV treatment increases the infiltration of the CD4^+^ and CD8^+^ T cells in the tumor microenvironment.

### 3.6. PRV Treatment Increased the PD-L1 Expression in the Tumor Microenvironment

**Figure 7:**
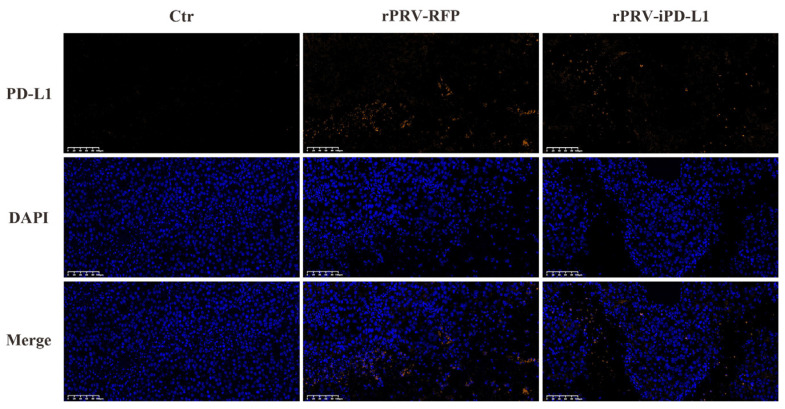
Immunofluorescence of PD-L1 in the tumor of each group. The image displayed represents one mouse from each group and is shown at 20× magnification (Scale bar = 100 μm).

To explore the potential mechanism behind the enhanced anti-tumor activity of rPRV-iPD-L1 compared to rPRV-RFP, we immunostained PD-L1 as it has been reported that oncolytic virus treatment induces upregulation of PD-L1 in tumor cells. Indeed, immunofluorescence analysis showed that the expression of PD-L1 in tumor areas in rPRV-RFP- and rPRV-iPD-L1-treated mice were much higher than that in the control group (Figure 7). PRV-induced upregulation of PD-L1 in tumor microenvironments may hamper T-cell-mediated killing of tumor cells, and the expression of iPD-L1 may counteract this inhibition, explaining the enhanced anti-tumor activity of rPRV-iPD-L1.

## 4. Discussion

We demonstrated in this study that PRV possesses a robust capacity to infect and kill tumor cells both in vitro and in vivo. Notably, the introduction of iPD-L1 in PRV significantly enhanced its anti-tumor efficacy in vivo. These findings suggest that PRV holds great promise as an oncolytic virus for treating tumors. Moreover, it has the potential to improve its anti-tumor effects by expressing immune modulators like iPD-L1, as demonstrated in this study.

PRV’s ability to infect a wide spectrum of tumor types of different species demonstrates its potential to be used in both animal and human cancer treatments. PRV is also a neuroinvasive herpesvirus, making it a potential cancer treatment for brain tumors, for example, glioblastoma. PRV exhibits the ability to infect a diverse array of tumor types across various species. We demonstrated its ability to infect and kill mouse tumor cells (B16-F10 and Panc02), canine mammary tumor cells (B-CMT), as well as many human tumor cells. This supports previous research indicating PRV’s capability to target both mouse and human cancer cells [25,34,35]. Previous studies have documented PRV’s infectivity in various human cancers, including neuroblastoma, glioblastoma, hepatoma, bladder cancer, colorectal cancer, and small-cell lung cancer. Additionally, PRV has been shown to infect mouse bladder cancer, pancreatic cancer, breast cancer, and colon cancer in vitro [36,37].

The broad spectrum of tumor types PRV can infect across different species underscores its potential for both animal and human cancer therapies. PRV’s neuroinvasive nature further positions it as a promising candidate for treating brain tumors, such as glioblastoma. In one study, an attenuated PRV derived from another vaccine strain, HB2000, was used to treat tumors grown from glioblastoma multiforme (GBM) cells, HepG2, and A498 in immune-deficient mice, demonstrating complete tumor clearance [25].

Further deletion of a pathogenicity-related gene from PRV Bartha enhances its safety. PRV Bartha is a naturally attenuated PRV, primarily due to the deletion of a genome segment containing the pathogenesis-causing genes *gE* and *gI*. Widely used as a vaccine in the global pig industry, this strain has not raised concerns regarding human safety. However, it has been shown to cause the death of mice [38]. One study demonstrated that Bartha inhibits the growth of human colorectal cancer in BALB/c nude mice, but all mice died on the 16th day of treatment [34]. Comparisons of the pathogenicity and anti-tumor effect of *TK*, *UL50*, or *US3* gene-deletion Bartha strains indicate that all three single-gene deletion strains increase safety in mice without sacrificing their anti-tumor activity, with the *TK* knockout strain showing the best result. Additionally, no abnormalities in the brains, lungs, and kidneys of treated mice were observed, nor were any significant changes in body weight in rPRV-RFP- or rPRV-iPD-L1-treated mice.

Consistent with our study, the deletion of *gE*/*gI*/*TK* from various variant PRV stains has also been demonstrated to significantly reduce pathogenicity in mice. Mice injected with 10^6^ TCID_50_ rPRV TJ-delgE/gI/TK showed no clinical signs and there was no detectable PRV DNA in the brain samples from the inoculated mice [39]. Another study demonstrated that mice inoculated with 10^6.0^ TCID_50_ rPRV NY-gE^−^/gI^−^/TK^−^ survived without any clinical symptoms and exhibited no pathological lesions in organs at 14 dpi [40]. Because variant PRVs are more pathogenic than classical PRVs, the Bartha *TK* knockout (rPRV-ΔTK) strain is likely even safer than the PRV variant strain rPRV-delgE/gI/TK as an oncolytic viral vector.

Immune checkpoint blockade (ICB) has emerged as a prominent method in cancer treatment; however, long-lasting positive responses are observed only in a subset of patients [41,42,43,44]. The effectiveness of this treatment is linked to pre-existing immune responses against the tumor, characterized by increased levels of tumor-infiltrating lymphocytes (TILs), a high mutation burden, and a broad neoantigen repertoire. Combining oncolytic viruses with ICB is an attractive approach, as oncolytic viruses have the potential to attract TILs into immune-compromised tumors by promoting the release of tumor antigens, danger signals, and proinflammatory cytokines [15,45]. These actions can subsequently bolster T cell recruitment and facilitate the activation of immune cells. Indeed, we observed enhanced infiltration of lymphocytes, including CD4^+^ and CD8^+^ T cells, within the tumor microenvironment following PRV treatment. 

Viral infections have been shown to elevate the expression of immune checkpoint molecules such as CTLA-4 and PD-L1 [46,47]. Our study reveals that PRV treatment increased PD-L1 expression in the tumor microenvironment. Normally, these molecules inhibit T cell activation, hindering the body’s anti-tumor immune response. However, this heightened expression sensitizes tumors to ICB [15]. Previous studies have demonstrated that oncolytic HSV-1 can remodel the pancreatic tumor microenvironment in an immunocompetent tumor model, significantly reducing tumor burden and prolonging the survival of tumor-bearing mice [48].

Arming oncolytic viruses with ICBs by encoding checkpoint inhibitors or antibodies to block PD-L1 or CTLA-4 within the virus itself may yield comparable or even better anti-tumor activity compared to combinations of an oncolytic virus and ICBs. This strategy has the potential to improve the therapeutic index of the ICBs by restricting their activity to the tumor microenvironment [49]. An engineered vaccine virus expressing GM-CSF and a PD-L1 inhibitor activates neoantigen-specific T cells, demonstrating enhanced anti-tumor activities, especially in cases resistant to PD-1/PD-L1 blockade therapy [40]. Consistent with previous reports, we demonstrate that the insertion of iPD-L1 into PRV improves anti-tumor activity compared to PRV alone [33,50].

## 5. Conclusions

PRV infects and lyses human, mouse, and dog cancer cells in vitro. rPRV-ΔTK exhibits a strong anti-tumor effect on the B16-F10 melanoma mouse model, which is further enhanced by carrying a PD-L1 inhibitor in rPRV-ΔTK. These results strongly support the promising role of this oncolytic rPRV-iPD-L1 as a novel agent in treating both human and animal cancers.

## Data Availability

The original contributions presented in the study are included in the article, further inquiries can be directed to the corresponding author.

## References

[1] Waldman A.D., Fritz J.M., Lenardo M.J. (2020). A guide to cancer immunotherapy: From T cell basic science to clinical practice. Nat. Rev. Immunol..

[2] Fan T., Zhang M., Yang J., Zhu Z., Cao W., Dong C. (2023). Therapeutic cancer vaccines: Advancements, challenges, and prospects. Signal Transduct. Target. Ther..

[3] Chan J.D., Lai J., Slaney C.Y., Kallies A., Beavis P.A., Darcy P.K. (2021). Cellular networks controlling T cell persistence in adoptive cell therapy. Nat. Rev. Immunol..

[4] Park J., Hsueh P.-C., Li Z., Ho P.-C. (2023). Microenvironment-driven metabolic adaptations guiding CD8+ T cell anti-tumor immunity. Immunity.

[5] Bagchi S., Yuan R., Engleman E.G. (2021). Immune Checkpoint Inhibitors for the Treatment of Cancer: Clinical Impact and Mechanisms of Response and Resistance. Annu. Rev. Pathol..

[6] Korman A.J., Garrett-Thomson S.C., Lonberg N. (2022). The foundations of immune checkpoint blockade and the ipilimumab approval decennial. Nat. Rev. Drug Discov..

[7] Zou W., Wolchok J.D., Chen L. (2016). PD-L1 (B7-H1) and PD-1 pathway blockade for cancer therapy: Mechanisms, response biomarkers, and combinations. Sci. Transl. Med..

[8] Jenkins R.W., Barbie D.A., Flaherty K.T. (2018). Mechanisms of resistance to immune checkpoint inhibitors. Br. J. Cancer.

[9] Zhu S., Zhang T., Zheng L., Liu H., Song W., Liu D., Li Z., Pan C.X. (2021). Combination strategies to maximize the benefits of cancer immunotherapy. J. Hematol. Oncol..

[10] Butterfield L.H., Najjar Y.G. (2024). Immunotherapy combination approaches: Mechanisms, biomarkers and clinical observations. Nat. Rev. Immunol..

[11] Zhu Z., McGray A.J.R., Jiang W., Lu B., Kalinski P., Guo Z.S. (2022). Improving cancer immunotherapy by rationally combining oncolytic virus with modulators targeting key signaling pathways. Mol. Cancer.

[12] Shalhout S.Z., Miller D.M., Emerick K.S., Kaufman H.L. (2023). Therapy with oncolytic viruses: Progress and challenges. Nat. Rev. Clin. Oncol..

[13] Ramelyte E., Tastanova A., Balázs Z., Ignatova D., Turko P., Menzel U., Guenova E., Beisel C., Krauthammer M., Levesque M.P. (2021). Oncolytic virotherapy-mediated anti-tumor response: A single-cell perspective. Cancer Cell.

[14] Wang L., Chard Dunmall L.S., Cheng Z., Wang Y. (2022). Remodeling the tumor microenvironment by oncolytic viruses: Beyond oncolysis of tumor cells for cancer treatment. J. Immunother. Cancer.

[15] Lin D., Shen Y., Liang T. (2023). Oncolytic virotherapy: Basic principles, recent advances and future directions. Signal Transduct. Target. Ther..

[16] Ma J., Ramachandran M., Jin C., Quijano-Rubio C., Martikainen M., Yu D., Essand M. (2020). Characterization of virus-mediated immunogenic cancer cell death and the consequences for oncolytic virus-based immunotherapy of cancer. Cell Death Dis..

[17] Gong T., Liu L., Jiang W., Zhou R. (2019). DAMP-sensing receptors in sterile inflammation and inflammatory diseases. Nat. Rev. Immunol..

[18] Tian Y., Xie D., Yang L. (2022). Engineering strategies to enhance oncolytic viruses in cancer immunotherapy. Signal Transduct. Target. Ther..

[19] Uche I.K., Fowlkes N., Vu L., Watanabe T., Carossino M., Nabi R., Del Piero F., Rudd J.S., Kousoulas K.G., Rider P.J.F. (2020). The Novel Oncolytic Herpes Simplex Virus Type-1 (HSV-1) VC2 Promotes Long-lasting, Systemic Anti-melanoma Tumor Immune Responses and Increased Survival in an Immunocompetent B16F10-derived Mouse Melanoma Model. J. Virol..

[20] Pomeranz L.E., Reynolds A.E., Hengartner C.J. (2005). Molecular biology of pseudorabies virus: Impact on neurovirology and veterinary medicine. Microbiol. Mol. Biol. Rev..

[21] Zheng H.-H., Fu P.-F., Chen H.-Y., Wang Z.-Y. (2022). Pseudorabies Virus: From Pathogenesis to Prevention Strategies. Viruses.

[22] Laval K., Vernejoul J.B., Van Cleemput J., Koyuncu O.O., Enquist L.W. (2018). Virulent Pseudorabies Virus Infection Induces a Specific and Lethal Systemic Inflammatory Response in Mice. J. Virol..

[23] Wang G., Zha Z., Huang P., Sun H., Huang Y., He M., Chen T., Lin L., Chen Z., Kong Z. (2022). Structures of pseudorabies virus capsids. Nat. Commun..

[24] Zhou M., Abid M., Cao S., Zhu S. (2023). Recombinant Pseudorabies Virus Usage in Vaccine Development against Swine Infectious Disease. Viruses.

[25] Wang G., Cao J., Gui M., Huang P., Zhang L., Qi R., Chen R., Lin L., Han Q., Lin Y. (2023). The potential of swine pseudorabies virus attenuated vaccine for oncolytic therapy against malignant tumors. J. Exp. Clin. Cancer Res..

[26] Xu A., Qin C., Lang Y., Wang M., Lin M., Li C., Zhang R., Tang J. (2015). A simple and rapid approach to manipulate pseudorabies virus genome by CRISPR/Cas9 system. Biotechnol. Lett..

[27] Zhang R., Chen S., Zhang Y., Wang M., Qin C., Yu C., Zhang Y., Li Y., Chen L., Zhang X. (2021). Pseudorabies Virus DNA Polymerase Processivity Factor UL42 Inhibits Type I IFN Response by Preventing ISGF3-ISRE Interaction. J. Immunol..

[28] Li R., Wu H., Sun Y., Zhu J., Tang J., Kuang Y., Li G. (2021). A Novel Canine Mammary Cancer Cell Line: Preliminary Identification and Utilization for Drug Screening Studies. Front. Vet. Sci..

[29] Cui D., Li L., Lou H., Sun H., Ngai S.M., Shao G., Tang J. (2014). The ribosomal protein S26 regulates p53 activity in response to DNA damage. Oncogene.

[30] Bo Z., Li X. (2022). A Review of Pseudorabies Virus Variants: Genomics, Vaccination, Transmission, and Zoonotic Potential. Viruses.

[31] Zhang R., Xu A., Qin C., Zhang Q., Chen S., Lang Y., Wang M., Li C., Feng W., Zhang R. (2017). Pseudorabies Virus dUTPase UL50 Induces Lysosomal Degradation of Type I Interferon Receptor 1 and Antagonizes the Alpha Interferon Response. J. Virol..

[32] Qin C., Zhang R., Lang Y., Shao A., Xu A., Feng W., Han J., Wang M., He W., Yu C. (2019). Bclaf1 critically regulates the type I interferon response and is degraded by alphaherpesvirus US3. PLoS Pathog..

[33] Wang G., Kang X., Chen K.S., Jehng T., Jones L., Chen J., Huang X.F., Chen S.-Y. (2020). An engineered oncolytic virus expressing PD-L1 inhibitors activates tumor neoantigen-specific T cell responses. Nat. Commun..

[34] Chai C., Zhang J., Zhou Y., Yin H., Zhang F., Diao Y., Zan X., Ma Y., Wang Y., Wu Y. (2022). The Effects of Oncolytic Pseudorabies Virus Vaccine Strain Inhibited the Growth of Colorectal Cancer HCT-8 Cells In Vitro and In Vivo. Animals..

[35] Han X., Sun J., Lv X., Tang X., Zheng Y., Ma J., Sun Y. (2023). A Recombinant Oncolytic Pseudorabies Virus Expressing Interleukin-18, Interferon-Gamma and PH20 Genes Promotes Systemic Antitumor Immunity. Microorganisms.

[36] Wollmann G., Tattersall P., van den Pol A.N. (2005). Targeting human glioblastoma cells: Comparison of nine viruses with oncolytic potential. J. Virol..

[37] Boldogkoi Z., Bratincsak A., Fodor I. (2002). Evaluation of pseudorabies virus as a gene transfer vector and an oncolytic agent for human tumor cells. Anticancer. Res..

[38] Brittle E.E., Reynolds A.E., Enquist L.W. (2004). Two modes of pseudorabies virus neuroinvasion and lethality in mice. J. Virol..

[39] Cong X., Lei J.L., Xia S.L., Wang Y.M., Li Y., Li S., Luo Y., Sun Y., Qiu H.J. (2016). Pathogenicity and immunogenicity of a gE/gI/TK gene-deleted pseudorabies virus variant in susceptible animals. Vet. Microbiol..

[40] Zhao Y., Wang L.-Q., Zheng H.-H., Yang Y.-R., Liu F., Zheng L.-L., Jin Y., Chen H.-Y. (2020). Construction and immunogenicity of a gE/gI/TK-deleted PRV based on porcine pseudorabies virus variant. Mol. Cell. Probes.

[41] Huang A.C., Zappasodi R. (2022). A decade of checkpoint blockade immunotherapy in melanoma: Understanding the molecular basis for immune sensitivity and resistance. Nat. Immunol..

[42] Wolchok J.D., Chiarion-Sileni V., Gonzalez R., Grob J.-J., Rutkowski P., Lao C.D., Cowey C.L., Schadendorf D., Wagstaff J., Dummer R. (2022). Long-Term Outcomes with Nivolumab Plus Ipilimumab or Nivolumab Alone Versus Ipilimumab in Patients with Advanced Melanoma. J. Clin. Oncol..

[43] Loo K., Smithy J.W., Postow M.A., Betof Warner A. (2021). Factors Determining Long-Term Antitumor Responses to Immune Checkpoint Blockade Therapy in Melanoma. Front. Immunol..

[44] Ravi A., Hellmann M.D., Arniella M.B., Holton M., Freeman S.S., Naranbhai V., Stewart C., Leshchiner I., Kim J., Akiyama Y. (2023). Genomic and transcriptomic analysis of checkpoint blockade response in advanced non-small cell lung cancer. Nat. Genet..

[45] Melcher A., Harrington K., Vile R. (2021). Oncolytic virotherapy as immunotherapy. Science.

[46] Kelly K.R., Espitia C.M., Zhao W., Wu K., Visconte V., Anwer F., Calton C.M., Carew J.S., Nawrocki S.T. (2017). Oncolytic reovirus sensitizes multiple myeloma cells to anti-PD-L1 therapy. Leukemia.

[47] Cai H., Liu G., Zhong J., Zheng K., Xiao H., Li C., Song X., Li Y., Xu C., Wu H. (2020). Immune Checkpoints in Viral Infections. Viruses.

[48] Zhang L., Wang W., Wang R., Zhang N., Shang H., Bi Y., Chen D., Zhang C., Li L., Yin J. (2020). Reshaping the Immune Microenvironment by Oncolytic Herpes Simplex Virus in Murine Pancreatic Ductal Adenocarcinoma. Mol. Ther. J. Am. Soc. Gene Ther..

[49] Harrington K., Freeman D.J., Kelly B., Harper J., Soria J.-C. (2019). Optimizing oncolytic virotherapy in cancer treatment. Nat. Rev. Drug Discov..

[50] Ju F., Luo Y., Lin C., Jia X., Xu Z., Tian R., Lin Y., Zhao M., Chang Y., Huang X. (2022). Oncolytic virus expressing PD-1 inhibitors activates a collaborative intratumoral immune response to control tumor and synergizes with CTLA-4 or TIM-3 blockade. J. Immunother. Cancer.

